# Characterizing the Effects of VPA, VC and RCCS on Rabbit Keratocytes onto Decellularized Bovine Cornea

**DOI:** 10.1371/journal.pone.0050114

**Published:** 2012-11-29

**Authors:** Ying Dai, Jiansu Chen, Hongyang Li, Shanyi Li, Jian Chen, Yong Ding, Jing Wu, Chan Wang, Meihua Tan

**Affiliations:** 1 Key Laboratory for Regenerative Medicine of Ministry of Education, Jinan University, Guangzhou, People's Republic of China; 2 Institute of Ophthalmology, Medical College, Jinan University, Guangzhou, People's Republic of China; 3 Ophthalmology Department, First Affiliated Hospital of Jinan University, Guangzhou, People's Republic of China; Instituto Butantan, Brazil

## Abstract

To investigate the morphological and growth characteristics of rabbit keratocytes when cultured on decellularized cornea under simulate microgravity (SMG) rotary cell culture system (RCCS) and static culture or in plastic culture supplemented with small molecules of valproic acid (VPA) and vitamin C (VC). Bovine corneas were firstly decellularized with Triton X-100 and NH_4_OH and through short-term freezing process. Then cell count kit-8 (CCK-8) and flow cytometry were used to test the effects of VPA and VC on the proliferation, cell cycle and apoptosis of rabbit keratocytes. Hematoxylin-eosin (H&E) staining and scanning electron microscopy (SEM) imaging showed that cells were eliminated in the decellularized bovine corneas. The proliferation of cultured keratocytes was promoted by VPA and VC in the cell proliferation assay. VPA and VC moderately decreased the number of apoptotic cells and obviously promoted cell-cycle entrance of keratocytes. Rabbit keratocytes in plastic displayed spindle shape and rare interconnected with or without VPA and VC. Cells revealed dendritic morphology and reticular cellular connections when cultured on the carriers of decellularized corneas supplemented with VPA and VC even in the presence of 10% fetal bovine serum (FBS). When cultured in RCCS supplemented with VPA, VC and 10% FBS, keratocytes displayed round shape with many prominences and were more prone to grow into the pores of carriers with aggregation. Reverse transcription-polymerase chain reaction (RT-PCR) analysis proved that the keratocytes cultured on decellularized bovine cornea under SMG with VPA and VC expressed keratocan and lumican. Keratocytes cultured on plastic expressed lumican but not keratocan. Immunofluorescence identification revealed that cells in all groups were positively immunostained for vimentin. Keratocytes on decellularized bovine cornea under SMG or in static culture were positively immunostained for keratocan and lumican. Thus, we reasonably made a conclusion that the combination of VPA, VC, RCCS and decellularized corneal carriers provide a good condition for keratocytes to well grow. Keratocytes can be manipulated to be aggregates or physiological morphological growth in vitro, which are important for the research of corneal stem cells and corneal tissue engineering.

## Introduction

Keratocytes account for 2.4% of the volume of corneal stroma, and they produce corneal extracellular matrix (ECM) and can keep the stability of ECM composition. Furthermore, keratocytes regulate the distribution of collagen fiber so that they are important to maintain the transparency and the tension of cornea [1]. Keratocytes can differentiate to fibroblast or myofibroblast phenotypes, which are controlled by specific environmental signals. The keratocyte differentiation plays an important role in retaining corneal transparency and determining the corneal response to injury [2]. Keratocytes may display different morphologic and phenotype changes with the changes in environmental conditions. Therefore, understanding the behaviors of keratocytes in different conditions will greatly increase our knowledge on corneal stromal function, corneal wound healing and regeneration [3]. Espana et al. [4] found that human keratocytes maintain dendritic morphology and keratocan expression when subcultured on human amniotic membrane (AM) stromal matrix even in the presence of 10% fetal bovine serum (FBS). Such a characteristic morphology can be achieved on plastic culture only in a serum-free medium, but is rapidly lost in a serum-containing medium. In our previous study, we have successfully cultured keratocytes on the surface of culture plates, AM, collagen–chitosan–sodium hyaluronate complexes, and so on [5], [6]. We also found that the growth and proliferation of rabbit keratocytes could be promoted by using dehydrated bovine acellular corneal stroma as carriers to culture rabbit keratocytes under simulate microgravity (SMG) of a rotary cell culture system (RCCS, Synthecon, Houston, TX, USA). The carriers of acellular corneal stroma could be obtained by dehydration of glycerol for 6 months, low-temperature preservation at −20°C or 4°C, and trypsinization for 10 min. After 19 days in SMG culture, rabbit keratocytes on the carriers were dendritic or spindle shaped and grew into the porous matrix of carriers [7]. In present study, we used a more rapid and convenient short-term chemical-frozen way to decellularize bovine cornea as carriers. At the same time, in order to observe the growing potential of keratocytes, we cultivated rabbit keratocytes on carries under SMG and static culture condition in culture medium supplemented with valproic acid (VPA) and vitamin C (VC). VPA is an inhibitor of DNA methyltransferase and histone deacetylase (HDAC) and can improve reprogramming efficiency of stem cells [8]. VC has been shown to increase the proliferative rate of cultured corneal fibroblasts and to stimulate the synthesis and secretion of appropriate ECM which comprises parallel arrays of ECM fibrils [9]. VC can also increase reprogramming efficiency by enhancing cell proliferation potential and alleviating cell senescence [10]. So, we expect to identify the effects of microenvironment and small molecules on rabbit keratocytes growth, and observe the characterizing changes of RCCS, VPA and VC on rabbit keratocytes onto decellularized bovine cornea.

## Materials and Methods

### Ethics Statement

Primary cultures were established from the corneas of New Zealand White rabbit (4 eyes) which were aged 3–4 months old with a weight range of 2–2.5 kg. Rabbits were handled in accordance with the ARVO Statement on the Use of Animals in Ophthalmic and Vision Research. The protocol was approved by the Institute Animal Care and Use Committee of Jinan University (Permit Number: 2011102101). All surgery was performed under sodium pentobarbital anesthesia, and all efforts were made to minimize suffering.

The bovine eyes were obtained at a local slaughter house (Shipai, Guangzhou, Guangdong, China) and their corneas were checked to be free of defects by slit lamp examination.

### Materials

Culture reagents were from Gibco (Grand Island, NY, USA). Corneal keratocytes were cultured in a complete growth medium that consisted of Dulbecco's Modified Eagle's Medium (DMEM), supplemented with 3.7 g/L NaHCO_3_, 100-U/mL penicillin G sodium, 100-mg/mL streptomycin sulfate, and 10% vol/vol FBS. Unless otherwise stated, all the other reagents were from Sigma (St. Louis, MO, USA). VPA was from Suju (Guangzhou, China). Cell Counting Kit-8 (CCK-8) was from Dojindo (Kyushu, Japan). Cell Cycle and Apoptosis Analysis Kit and Annexin V-FITC/PI apoptosis detection kit were from KeyGEN (Nanjing, China). Monoclonal anti-vimentin (NeoMarkers) was from Lab Vision Corp (Fremont, MO, USA). Goat anti-keratocan polyclonal antibody, goat anti-lumican polyclonal antibody and goat anti-rabbit IgG were from Santa Cruz Biotechnology (Santa Cruz, CA, USA). EZgene™ Tissue RNA Miniprep Kit was from Biomiga (San Diego, CA, USA). ReverTra Ace qPCR RT Kit, Blend Taq® and Blend Taq®-Plus were from Toyobo (Osaka, Japan). Primers were synthetized by BGI (Beijing, China).

### Methods

#### Preparation of short-term chemical-frozen decellularization of bovine stromal carriers

Fresh bovine eyes were obtained and the cornea was excised, rinsed with saline containing antibiotic solution (prepared with 100-U/mL penicillin G sodium and 100-mg/mL streptomycin sulfate), and dissected under sterile condition. Bovine stromal lamella (1 mm thick) was removed, treated with 0.5% Triton X-100 and 20 mM NH_4_OH mixture for 5–10 min. After rinsed with phosphate buffered saline (PBS) three times, bovine stromal lamellas were frozen in −80°C for 3 d and then preserved in 100% glycerol at 4°C. Prior to use, the dehydrated bovine stroma was rehydrated in PBS. Then, the stroma was cut into pieces (3×3×1 mm, n = 30) as cell carriers and sterilized under ultraviolet light for 30 min.

#### Histology analysis

Decellularizated bovine cornea and normal bovine cornea were obtained for hematoxylin-eosin (H&E) staining under light microscopy. The staining was as follows: The samples were put in 95% ethanol for 15 min after being rinsed with PBS buffer three times, washed with tap water twice for 1 min each time, stained in small amounts of hematoxylin for 1 min, and again rinsed with tap water. The samples were soaked in dilute hydrochloric acid for 5 min and were then rinsed with tap water. After that, they were put in dilute ammonia water for 3–5 min and then rinsed again. As a final step, the samples were stained in eosin for 5–10 min and then rinsed with tap water. Samples were cut into 5-µm-thick sections by freezing microtome, stained with H&E, examined, and photographed using a photomicroscope.

#### Isolation and primary culture of keratocytes

Eyes from New Zealand White rabbits were obtained and cornea was excised for keratocytes. Connective tissue and external muscles were then removed. The corneas were rinsed with saline containing antibiotic solution. In this study, we adopted the culture procedures of keratocytes of Jester et al [11]. Briefly, corneas stripped of both endothelial and epithelial tissues were put in a solution of 0.2% type I collagenase in culture medium overnight at 37°C. The keratocytes were then rinsed in the culture medium composed of DMEM, centrifuged (200×g, 5 min), and suspended at a concentration of 1×10^4^ cells/mL in a culture medium supplemented with 10% FBS. The cells were seeded into a 25 cm^2^ plastic culture flask, fed with 4 mL of media, and incubated at 37°C in a 5% CO_2_ incubator. The culture medium was changed every second day.

#### Cell proliferation assay

A cell count kit-8 (CCK-8) was employed to identify the effect of VPA and VC on the proliferation of keratocytes on the decellularizated bovine cornea (group 1) and plastic (group 2). 1×10^4^ cells were seeded and cultured at 37°C for 24 h. Then the culture medium was removed. Subsequently, cells were treated with or without 1 mM VPA and 50 ug/ml VC in the presence of 10% FBS for a further 72 h. After 10 ul dye was add to each well, cells were incubated at 37°C for 2 h. The absorbance at 450 nm was determined using multimode reader. Six parallel experiments in each sample were used to assess the cell proliferation.

#### Flow cytometry

To determine the cell cycle of keratocytes within the culture solution with or without VPA and VC, flow cytometry was performed. Keratocytes were cultured in media in 6-well culture plates with or without 1 mM VPA and 50 ug/ml VC for 3 days. Cells from the two groups were resuspended in cold alcohol and then stored at −4°C overnight before flow cytometry analysis. Data analysis was conducted using ModFit software.

VPA and VC were used to test whether VPA and VC have an anti-apoptotic effect in cultivated keratocytes. The method was performed as manufacture instructions and as previously described [12]. Keratocytes were cultured in media in 6-well culture plates with or without 1 mM VPA and 50 ug/ml VC for 3 days till cells were about 60–70% confluent. Then media was replaced and cells were challenged with 125 µM H_2_O_2_ for another 12 h. Cells were harvested and washed in cold PBS. Keratocytes were treated with Annexin V-FITC/PI and then resuspended in binding buffer at a concentration of 1×10^6^ cells/ml. Data analysis was conducted using WinMDI software.

#### Keratocytes cultured under SMG

There were four groups in the experiment: static culture on plastic without VPA and VC as control A; static culture on plastic with VPA and VC as control B; static culture on carriers with VPA and VC as control C; SMG culture on carriers with VPA and VC as SMG experiment group.

Keratocytes cultured in a 6-well plate at a density of 1×10^4^ cells/mL under conventional condition with or without 1 mM VPA and 50 ug/ml VC were used as control A and control B, respectively. In control C, keratocytes were cultured on carriers in a 6-well plate at a concentration of 1×10^4^ cells/mL in the culture medium supplemented with 10% FBS, 1 mM VPA and 50 ug/ml VC under static condition. Then, cells were incubated at 37°C in a 5% CO_2_ environment. Culture medium was changed every second day.

In SMG experiment group, primary keratocytes were trypsinized and suspended at 5×10^5^ cells/mL. The prepared bovine stromal carriers (n = 30) were put into the cellular suspension, mixed gently, then cultured at 37°C in a 5% CO_2_ incubator for 30 min. Keratocytes and carriers were then cultured in the SMG culture system. The first step was to inject slowly 10 mL serum-free DMEM into the 25 mL capacity vessel of RCCS. Then, DMEM with keratocytes and carriers was put into this vessel. At last, DMEM was filled into the vessel at a concentration of 1×10^4^ cells/mL in the culture medium supplemented with 10% FBS, 1 mM VPA and 50 ug/ml VC. Gas bubbles in the RCCS vessel must be removed. The vessel was put into the incubator and rotational speed was set at 10, 15 and 20 rpm in the first, third, seventh day of culture, respectively. Culture samples were obtained from the carriers every 3 days for examination of cell morphology.

#### Reverse transcription–polymerase chain reaction (RT-PCR) analysis

Total RNA from keratocytes of four groups was isolated using Tissue RNA Miniprep Kit, and the resulting RNA samples were quantified by measuring the OD at 260 nm; the OD 260/280 ratios for all RNA samples were between 1.8 and 2.1. Total RNA (1 µg) was reverse transcribed in a 10 µl reaction mixture containing 2 µl 5× RT Buffer, 0.5 µl RT Enzyme Mix, 0.5 µl Primer Mix, 6 µl nuclease-free water at 42°C for 1 h. One tenth of the RT product was used for subsequent PCR with the final concentration of PCR reaction being 1× Buffer, 0.2 mM dNTPs, 1.25 U Blend Taq® in a total volume of 50 uL, using primers shown in Table 1. The PCR mixture was first denatured at 94°C for 2 min then amplified for 30 cycles (94°C, 30 sec; Tm-5°C, 30 sec; 72°C, 1 min) using a authorized thermal cycler (Eppendorf, Hamburg, GER). After amplification, 5 uL of each PCR product and 1 uL of 6× loading buffer were mixed and electrophoresed on a 1.5% agarose gel in 0.5× Tris-boric acid-EDTA containing 0.5 ug/mL ethidium bromide. Gels were photographed and scanned.

**Table 1:** List of primers.

Primers		Sequences (5′to 3′)	Size of PCR Product (bp)	GeneBank Accession Number
GAPDH	Sense	AGGTCATCCACGACCACTTC	202	NM_001082253
GAPDH	Antisense	GTGAGTTTCCCGTTCAGCTC		
Keratocan	Sense	CAAGACTGCCAGCCAATACA	206	XM_002711384
Keratocan	Antisense	GACCTTTGTGAGGCGATTGT		
Lumican	Sense	TGCAGCTTACCCACAACAAG	176	NM_001195680
Lumican	Antisense	AGGCAGTTTGCTCATCTGGT		

#### Immunofluorescence assay

Immunofluorescence was used to identify the rabbit keratocytes at day 8 of culture. To keep the distribution on carriers and growing morphology of keratocytes under SMG, we directly conducted fluorescent staining without cellular trypsinization. Briefly, after fixation in 4% paraformaldehyde for 30 min at room temperature, keratocytes were permeabilized with 0.1% Triton X-100 in PBS for 15 min at room temperature, washed three times with PBS and incubated with PBS containing 10% FBS for 30 min at room temperature. The cells were incubated with the monoclonal anti-vimentin (1∶500), goat anti-keratocan polyclonal antibody (1∶400) and goat anti-lumican polyclonal antibody (1∶400) for 60 min, and then with the secondary antibodies for 60 min at room temperature. The cells were rinsed with PBS twice for 3 min each time. Then, the samples were incubated in the moist chamber for 15 min with DAPI for nuclear stain. At last, the samples were washed again. The cells were examined by fluorescence microscope.

#### Scanning electron microscopy

Scanning electron microscopy (SEM) was used to observe the ultrastructure of the surface of the cells and carriers or the growing morphology of keratocytes. Samples were fixed in 2.5% glutaraldehyde, washed three times for 30 min each time in 0.1 M PBS, and then postfixed in 1% osmium tetroxide for 30 min. Samples were washed three times again in PBS before passing through a graded series of alcohol (50, 70, 80, 90, and 100%). After three 5-min changes of 100% ethanol, the samples were then transferred to isoamyl acetate for 30 min, critical point dried, coated with gold, and mounted for viewing in the JSM-T300 SEM (JEOL Technics Co. Ltd., Tokyo, Japan).

#### Statistical analysis

The values were expressed as means ± SD from three to six samples. Statistical analyses were carried out using Student's t test and a one-way analysis of variance (SPSS 16.0, Inc., Chicago, IL, USA). Results of p<0.05 were considered statistically significant.

## Results

### The observation of decellularized bovine cornea

**Figure 1:**
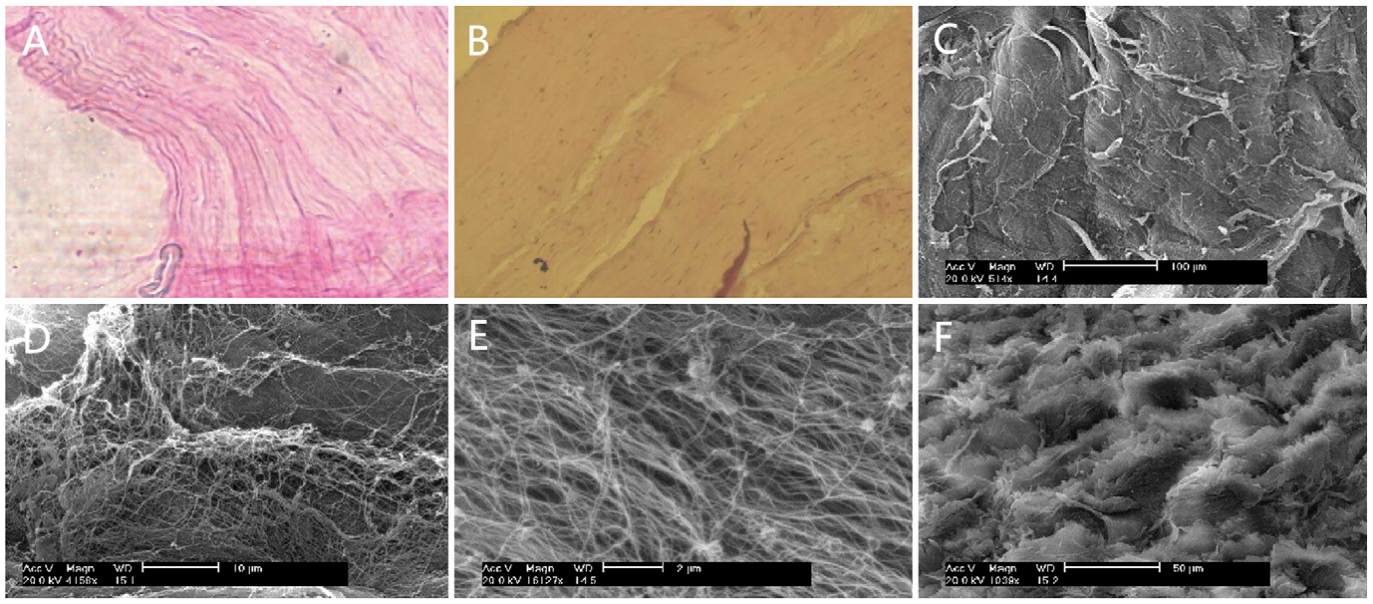
The morphological feature of decellularized bovine stroma. H&E staining showed that there were no cells in our decellularizated bovine cornea (A), while there were many keratocytes in the normal bovine corneal stroma (B), taken at ×400, ×200 magnification, respectively. SEM evaluated bovine acellular stromal lamella at the lower magnification (C) and higher magnifications (D, E) showed rough surfaces were rich in fibers and there were microporous structure. The cross section of acellular stroma had rough surface constituted by abundant lamellar structure (F). Acc. V, accelerative voltage; Magn, magnification; WD, work distance.

H&E staining showed that cells were eliminated in our decellularizated bovine cornea (Figure 1 A), while there were many keratocytes in the normal bovine corneal stroma (Figure 1 B). SEM evaluation indicated the rough surfaces of bovine acellular stromal lamella, which were composed of a series of fibers and shallow pores at the lower magnification (Figure 1 C) as well as collagen fibers arrayed regularly parallel and formed microporous structure at the higher magnifications (Figure 1 D, E). The cross section of acellular stroma had rough surface constituted by abundant lamellar structure (Figure 1 F). This result revealed that the cells of bovine cornea were removed by using our short-term chemical-frozen decellularization.

### The effects of VPA and VC on the proliferation, cell cycle and apoptosis of rabbit keratocytes

**Figure 2:**
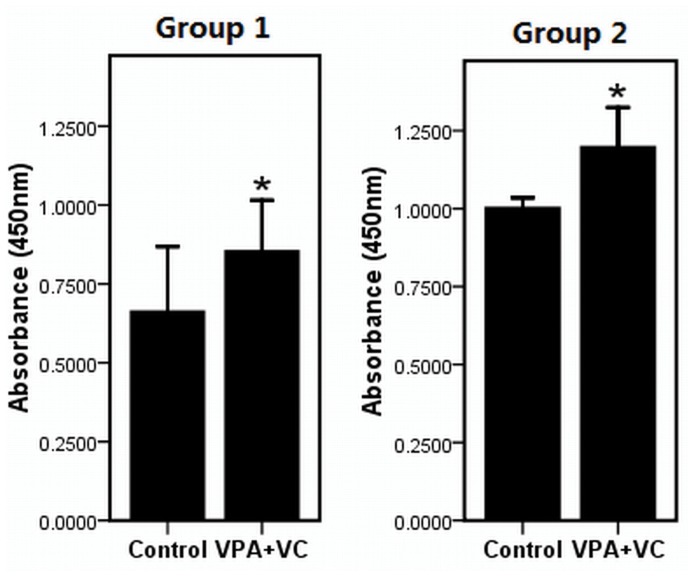
Keratocytes proliferation assay treated with or without VPA and VC. Cell proliferation in keratocytes with VPA and VC was detected by CCK-8 analysis. Cells were seeded on the carriers (group 1,) and plastic plate (group 2,). Difference with P<0.05(*) was considered statistically significant.

**Figure 3:**
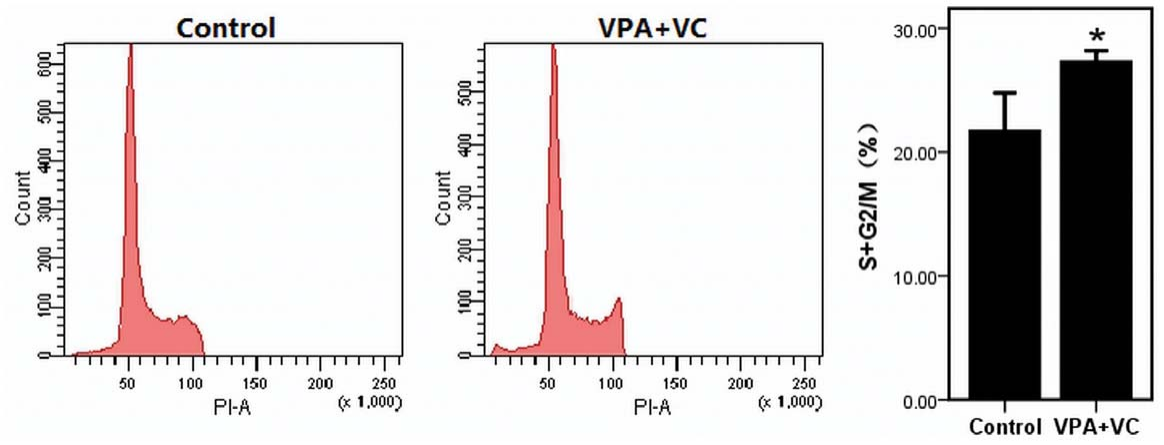
The cell-cycle assay of keratocytes with or without VPA and VC. 1 mM VPA and 50 ug/ml VC promoted cell-cycle entrance of keratocytes. Difference with P<0.05(*) was considered statistically significant.

**Figure 4:**
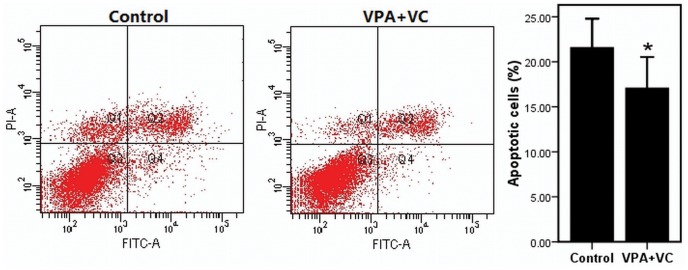
The effects of VPA and VC on anti-apoptosis of cultured keratocytes by stained with AnnexinV-FITC/Propidium Iodide. Keratocytes challenged with H_2_O_2_ showed (21.50±1.32)% apoptotic cells, whereas keratocytes with VPA and VC under the same challenge displayed (17.00±1.41)% apoptotic cells. Difference with P<0.05(*) was considered statistically significant.

The proliferations of keratocytes on the decellularizated bovine cornea (group 1) or plastic (group 2) were significantly promoted when supplemented with 1 mM VPA and 50 ug/ml VC based on CCK-8 assay (Figure 2). The cell-cycle entrance of keratocytes treated with 1 mM VPA and 50 ug/ml VC was significantly higher than keratocytes of control group without VPA and VC. The percentage of cells entering the S phase and G2/M phase in the VPA and VC group and control group were (27.23±0.38)% and (21.63±1.25)% respectively (Figure 3). Annexin V and PI were analyzed by flow cytometry to detect apoptosis in cultured keratocytes. Keratocytes challenged with H_2_O_2_ showed (21.50±1.32)% apoptotic cells, whereas keratocytes added 1 mM VPA and 50 ug/ml VC under the same challenge displayed (17.00±1.41)% apoptotic cells (Figure 4). The result showed that VPA and VC were able to reduce the ratio of apoptotic keratocytes challenged with H_2_O_2_.

### The observation of keratocytes by light microscopic evaluation

**Figure 5:**
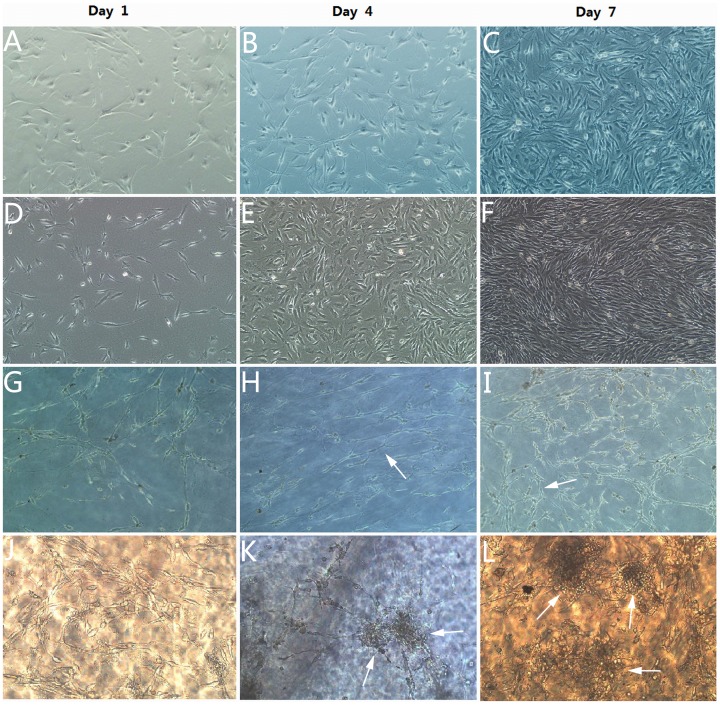
Light microscopic evaluations of keratocytes cultured in different conditions at day 1, day 4 and day 7. Keratocytes on plastic in static culture (A–C: without VPA and VC; D–F: with VPA and VC): spindle shape and rare interconnected at day 1, 4 and 7. Keratocytes on carriers in static culture (G–I): reticular structure formation at day 1 (G); arrows indicated regular and paralleled reticular structure at day 4 (H); arrows indicated richer cellular interconnection and reticular structure at day 7 (I). Keratocytes on carriers under SMG culture (J–L): smaller ellipse shape and reticular structure formation at day 1 (J); arrows indicated 3D aggregates at day 4 (K); arrows indicated more obvious spherical aggregation at day 7 (L). All photographs were taken at ×100 magnification.

In the presence of 10% FBS, almost all keratocytes on plastic in static culture without VPA and VC (control A) (Figure 5 A, B, C) and with VPA and VC (control B) (Figure 5 D, E, F) showed spindle shape, and rare irregularly interconnected or unconnected with each other. However, also 10% FBS in existence, keratocytes on the carriers of acellular bovine cornea in static culture with VPA and VC (control C) well adhered to carriers and interconnected to form reticular structure at day 1 of culture (Figure 5 G). The reticular structure became more regular and paralleled at day 4 of culture (Figure 5 H), and cells grew into dendritic shape and there were rich cell protrusions and interconnection at day 7 of culture (Figure 5 I). In the same presence of VPA, VC and 10% FBS, keratocytes on the same carriers of acellular bovine cornea under SMG culture showed smaller ellipse shape and formed reticular structure at day 1 (Figure 5 J). Discriminatively, at day 4 of SMG culture, a large number of keratocytes interconnected and formed three-dimensional (3D) aggregates, which was a distinctive phenomenon in SMG experiment group (Figure 5 K). Further, the spherical aggregation and proliferation of keratocytes became larger and more obvious at day 7 of SMG culture (Figure 5 L).

### Keratocan and lumican mRNA expression

**Figure 6:**
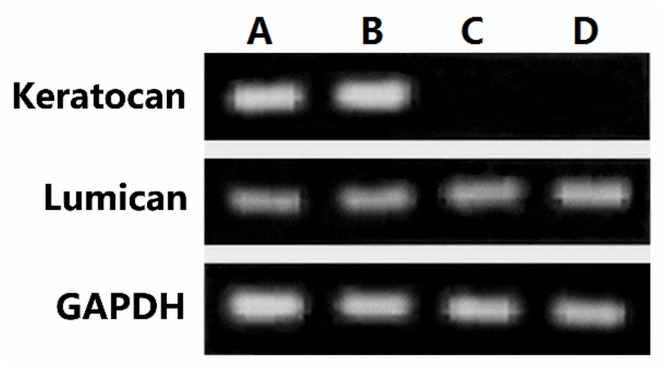
RT-PCR analysed the expression of keratocan and lumican. RT-PCR was used to determine the expression of keratocan (206 bp), lumican (176 bp) with GAPDH (202 bp) as an internal control in keratocytes at day 8 of culture. Keratocytes cultured on the carriers of acellular bovine cornea under SMG (A) or on the carriers in static condition (B) expressed abundant amounts of keratocan transcript. However, keratocan not expressed in cells cultured on plastic with or without VPA and VC (C, D). Lumican and GAPDH were expressed in the cells from all groups.

Total RNA was extracted from keratocytes of four groups at day 8 of culture, and RT-PCR was used to determine the expression of keratocan, lumican and GAPDH transcript with size of 206 bp, 176 bp and 202 bp. Keratocytes cultured on the carriers of acellular bovine cornea under SMG (Figure 6 A) or on the carriers in static condition (Figure 6 B) expressed abundant amounts of keratocan transcript. However, keratocan mRNA could not be detected in cells continuously cultured on plastic in DMEM containing 10% FBS with or without VPA and VC (Figure 6 C, D). Lumican and GAPDH mRNAs were expressed in the cells from all groups (Figure 6).

### Immunofluorescence identification

**Figure 7:**
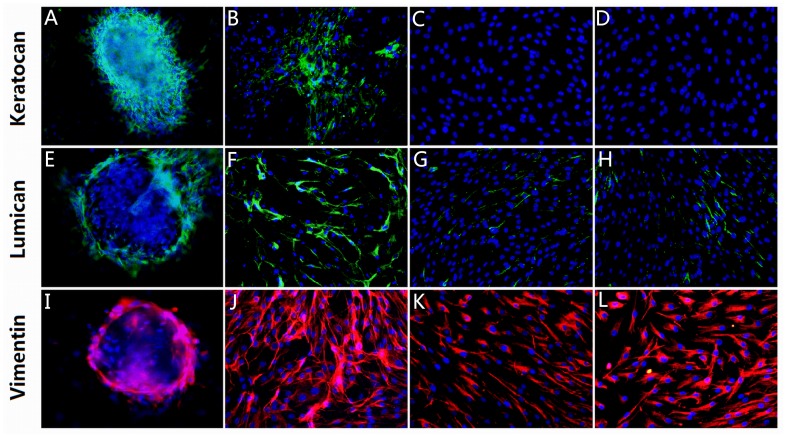
The immunofluorescence stains of keratocan, lumican and vimentin for keratocytes in four groups at day 8 of culture. Keratocytes at day 8 of SMG culture on the carriers richly interconnected to form 3D cell clumps and were positively immunostained for keratocan and lumican (A, E). Keratocytes on the carriers in static culture were positively immunostained for keratocan and lumican (B, F). Few cells in plastic with or without VPA and VC were positively immunostained for lumican (G, H). Keratocytes from four groups were positively immunostained for vimentin, which revealed that the cytoplasms were stained red, the nuclei were stained blue (I–L). All photographs were taken at ×200 magnification.

Keratocytes at day 8 of SMG culture on the carriers richly interconnected to form 3D cell clumps and were positively immunostained for keratocan and lumican (Figure 7 A, E). Keratocytes on the carriers in static culture were positively immunostained for keratocan and lumican (Figure 7 B, F). Few cells in plastic with or without VPA and VC were positively immunostained for lumican (Figure 7 G, H). Keratocytes from four groups were positively immunostained for vimentin, which revealed that the cytoplasms were stained red, the nuclei were stained blue (Figure 7 I–L).

### The observation of keratocytes by SEM evaluation

**Figure 8:**
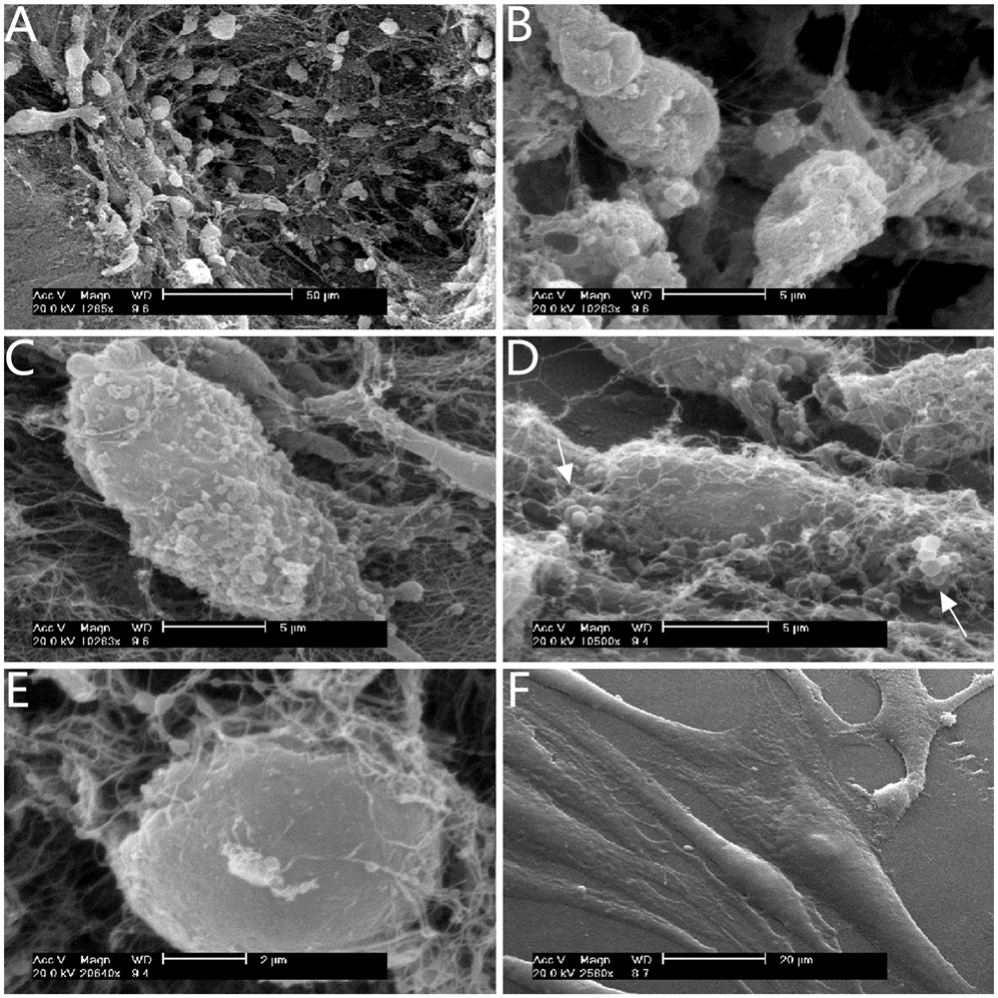
SEM images keratocytes at day 4 of culture. The growth of keratocytes under SMG (A–D): the cellular aggregation in a pore of the carrier (A); round shape and 3D interconnection of cells (B); cells with rough surfaces rich in globular prominences (C); cells seemed to be able to grow into reticular fibers of the carriers and arrows indicated pieces of beaded granular secretion (D). The growth of keratocytes on the carriers in static culture (E): rich interconnection with the carriers but relatively smooth cellular surfaces. The growth of keratocytes in plastic (F): spindle shape with flat and smooth surfaces of cells. Acc. V, accelerative voltage; Magn, magnification; WD, work distance.

At day 4 of culture, the cultured samples were taken to image by SEM. Keratocytes under SMG were prone to grow into the pores of the carriers with aggregated gathering growth (Figure 8 A). Cells displayed round and 3D interconnection (Figure 8 B). Single cell showed rough cellular surfaces which were rich in globular prominences (Figure 8 C). Keratocytes seemed to be able to grow into reticular fibers of the carriers and showed pieces of beaded granular secretion (Figure 8 D). Keratocytes on the carriers in static culture also showed rich interconnection with the carriers but cellular surfaces were relatively smooth (Figure 8 E). While keratocytes in plastic were spindle shape and their small cellular nucleus were easily displayed. In addition, flat cells were two-dimensional interconnection with plastic plates and cellular surfaces were smooth (Figure 8 F).

## Discussion

HDAC inhibitors are associated with epigenetic regulation and have neuroprotective effects under various neurodegenerative conditions. As a HDAC inhibitor, VPA is neuroprotective and can delay spontaneous cell death in purified rat retinal ganglion cells [13]. VPA also can significantly improve iPS reprogramming efficiency and the cloning efficiency of somatic cell nuclear transfer embryos [8], [14]. VC is an antioxidant and widely distribute in organisms [15]. Anti-oxidative vitamins can prevent apoptosis. Moteki et al. [16] reported that VC induced DNA synthesis and cell proliferation in primary cultures of adult rat hepatocytes by interacting with the IGF-I receptor site and by activating the receptor tyrosine kinase/MAP kinase pathway. VC also enhanced the growth as well as the production and secretion of type I and type III collagen peptid of the cultured rabbit keratocytes [17], [18]. Similarly in this study, we found that VPA and VC enhanced the viability and proliferation of rabbit keratocytes, increased upon stimulation of cells to enter the cell cycle and reduced the percentage of apoptotic cells after H_2_O_2_ treatment. As we all know, keratocytes are derived from the neural crest. We presume that above effects of VPA and VC on rabbit keratocytes partly from the protection by VPA and partly from the anti-apoptosis by VC [19].

The rotary bioreactors could influence major cellular events such as differentiation, proliferation, viability and cell cycle. Introduced by the National Aeronautics and Space Administration (NASA) of the USA in 1987, SMG cultures seem to be ideal for overcoming some drawbacks associated with static culturing systems [20], [21]. For instance, SMG conditions allow the cells to well proliferate in a RCCS of microgravity environment but at low shear stress and low turbulence environment. SMG culture conditions can provide appropriate microenvironments which have proven advantageous for intercellular communication on tissue-specific cell assembly, cell adhesion, signal transduction, glandular structures and function [22], [23]. In addition, when cells are maintained in a 3D growth environment they tend to aggregate. SMG culture can enhance cell-cell interactions and supply such 3D growth microenvironment. So, the aggregating growth of cells is an important effect of SMG on some cell lines [24]. SMG promoted porcine liver cells to grow into 3D cell aggregation, which displayed that SMG culture system was suitable for long-term and expanding of cell culture [25]. The dynamic flow of RCCS might improve nutrient supply and increase metabolic waste removal for the cells in the interior cellular spheres. Thus, comparison with the static culture, rotating simulated microgravity culture environment can show better cellular vitality and function for some cultivated cells. Our previous study found that SMG cultivation was propitious to proliferation of keratocytes for a rather long period of time [7]. Furthermore, SMG conditions are beneficial to culture stem cells. Studies revealed that NASA-approved rotary bioreactors promoted the proliferation and viability of epidermal stem cells and periodontal ligament stem cells. Epidermal stem cells cultured in RCCS inclined to aggregate on the microcarriers and form multilayer 3D epidermis-like structures [26], [27]. It was reported that SMG culture system was able to realize feeder-free and serum-free mouse embryonic stem cell cultivation without leukemia inhibitory factor [28]. SMG conditions were helpful for stem cells to keep proliferation at an undifferentiated state, embryos body formation and three germinal layer differentiations [29], [30].

Various types of biological materials or synthetic carriers had been widely used in cell culture and tissue engineering. When compared with wholly synthetic biomaterials, natural biomaterials are more promising because of the correct anatomical structure, flexibility, and suitable physical and mechanical properties [31]. Therefore, natural biomaterials such as ECM always serve as scaffold carriers for cell attachment, migration, and proliferation during cell culture and reconstruction of tissues [32], [33]. Carriers are pivot for somatic and stem cellular growth under SMG culture, especially for microgravity tissue engineering. Native biomaterials are more suitable for cell survival in microgravity environment. However, many decellularization processes from corneas usually need a long term and a series of complicate physical and chemical protocols. Multifarious and long treatments for decellularization are not so good for maintaining the properties of natural matrixes. In most cases, the highly organized structure of the corneal collagen fibers becomes impaired after the decellularization process, with partial elimination of some key extracellular matrix components such as proteoglycans [34]. Our previous study showed that bovine acellular stromal lamella appeared in favor of rabbit keratocyte growth in SMG condition. But the preparation process of acellular stromal lamella was rather long time, which underwent the dehydrated procedures of low-temperature preservation in glycerol for 6 months [7]. So, we here adopted short-term chemical-frozen to decellularizate bovine cornea and then prepared them as carriers to culture keratocytes. These native biological carriers have several advantages. Firstly, the whole decellularizated process carried out in three days. Short-term and simple chemical-frozen treatment could efficiently decellularize bovine corneal carriers and greatly saved time compared to those methods as described before. Secondly, the short-term chemical-frozen decellularization of bovine cornea was able to produce the rough surfaces of acellular stroma composed of a series of fibers arrayed regularly parallel and pores. Keratocytes onto these rough surfaces of carriers displayed dendritic shape and could interconnect to grow into regular reticular structure when with small molecules of VPA and VC as well as 10% FBS, which is very close to the native growing status of keratocytes in the normal cornea. The dendritic shape of keratocytes can be achieved on plastic culture in a serum-free medium. But usually it is difficult to obtain the reticular connection of keratocytes in plastic in serum-free condition. We also found that keratocytes on smooth surface of plastic with or without VPA and VC in the presence of 10% FBS just showed spindle shape and rare irregularly interconnected or unconnected with each other. Thus, we can speculate that the characteristic structure of dendritic shape and reticular arrangement of keratocytes comes mainly from decellularized corneal carriers in the presence of 10% FBS. Keratocytes in en face section of normal cornea comprise a network of broad cells that are interconnected by cellular processes [35]. Therefore, decellularized corneal carriers, VPA and VC are beneficial for maintaining normal keratocyte morphology. For the consideration of clinical applications, the decellularized human corneas and human keratocytes may be more conducive as they are closer to the native cornea. The human corneas are unsuitable for keratoplasty are adaptable candidates for applications. The recent studies showed that decellularized human cornea provided a scaffold that could support the growth of corneal epithelial cells and stromal fibroblasts. When exposed to corneal stroma, the corneal fibroblasts appeared to revert back into a more keratocyte-like phenotype [36]. The cells expressed high levels of keratocyte phenotypes no matter that human keratocytes were seeded on or injected into the stroma of decellularized cornea [34], [36], [37]. We suppose that decellularized corneal carriers, VPA and VC could be helpful for establishment of normal keratocyte morphology and network when keratocyte engraftments are considered.

Thirdly, the short-term treatment of decellularization did not significantly disrupt the corneal ECM structure, which easily formed more suitable surfaces for cells growth under SMG culture when compared to common artificial carriers such as Cytodex (data not shown). When under SMG culture condition supplemented with 10% FBS and small molecules of VPA and VC, keratocytes on acellular corneal carriers first also displayed cellular reticular formation as in static culture. But interestingly, later cellular gathering growth occurred and the 3D spherical aggregating growth of keratocytes became larger with time, which was different from the morphologic changes in static culture with the same environment of VPA, VC, FBS and carriers. Keratocytes showed rough surfaces rich in globular prominences. Keratocytes were able to grow into reticular fibers and showed pieces of beaded granular secretion. There were rich cell interconnections. All of these demonstrated that the combination of VPA, VC, RCCS and decellularized corneal carriers provided a good condition for keratocytes to well survive and actively function.

In this study, we found that VPA and VC promoted the proliferation and cell-cycle entrance of rabbit keratocytes, and decreased the numbers of apoptotic keratocytes. We used a more rapid and convenient way to decellularize bovine cornea as carriers. Keratocytes could show different morphologic changes in different environmental conditions. Our study confirmed that rabbit keratocytes maintained dendritic morphology and reticular cellular connections when cultured on bovine decellularized corneal stromal matrix supplemented with VPA and VC even in the presence of 10% FBS. If without using decellularized cornea as scaffold, even supplemented with VPA and VC, rabbit keratocytes in plastic could not display such a growing property. But when cultured in RCCS supplemented with VPA, VC and 10% FBS, keratocytes displayed round shape with many prominences and were more prone to grow into the pores of carriers with aggregation. Our previous study indicated that rabbit keratocytes on dehydrated bovine corneal stroma displayed spherical, dendritic or spindle shape for 19 days in RCCS and flat and spindle shaped in static culture in the presence of 10% FBS. So, from our previous and present data, we presume that supplement with VPA and VC are helpful for round morphologic and aggregating growth of keratocytes on decellularized cornea under SMG, and dendritic morphology and reticular cellular connections of keratocytes on decellularized cornea in static culture even 10% FBS in existence. These phenomena displayed that our decellularizated carriers provided natural microenvironment for 3D growth under SMG culture or native growing morphology of keratocytes under static culture. This study expects to lay foundation for the manipulation of keratocytes in vitro to be aggregative sphere or physiological morphological growth, which are important for corneal tissue engineering and corneal stem cell research.
